# Sirtuin 3 Protects Lung Adenocarcinoma from Ferroptosis by Deacetylating and Stabilizing Mitochondrial Glutamate Transporter Solute Carrier Family 25 Member A22

**DOI:** 10.3390/antiox14040403

**Published:** 2025-03-28

**Authors:** Xiangyun Wei, Tiange Wang, Zhengcao Xing, Qinyun Shi, Jianmin Gu, Qiuju Fan, Hao Wang, Bin Chen, Jinke Cheng, Rong Cai

**Affiliations:** 1 Department of Biochemistry & Molecular Cell Biology, Shanghai Jiao Tong University School of Medicine, Shanghai 200025, China; wxy18759692832@sjtu.edu.cn (X.W.); skywow@sjtu.edu.cn (T.W.); xzc1995@shutcm.edu.cn (Z.X.); gardenofforkingpath@sjtu.edu.cn (Q.S.); fanqiuju93@shsmu.edu.cn (Q.F.); jkcheng@shsmu.edu.cn (J.C.); 2 Department of Thoracic Surgery, Zhongshan Hospital, Fudan University, Shanghai 200025, China; gu.jianmin@zs-hospital.sh.cn (J.G.); wang.hao@zs-hospital.sh.cn (H.W.); 3 Department of Oncology, Shanghai Pulmonary Hospital, Tongji University School of Medicine, Shanghai 200433, China; binchen629@tongji.edu.cn

**Keywords:** lung adenocarcinoma, ferroptosis, Sirtuin 3, solute carrier family 25 member 22

## Abstract

Solute carrier family 25 member A22 (SLC25A22) is a glutamate transporter in the inner mitochondrial membrane that is known to suppress ferroptosis in pancreatic ductal adenocarcinoma (PDAC). Sirtuin 3 (SIRT3) is the main mitochondrial deacetylase, and we previously demonstrated that targeting SIRT3 sensitized glioblastoma to ferroptosis by promoting mitophagy and inhibiting SLC7A11. The purpose of this study was to analyze the effect of SIRT3-mediated deacetylation of mitochondrial SLC25A22 on RAS-selective lethal 3 (RSL3)-induced ferroptosis in lung adenocarcinoma (LUAD). We found that the expression of SLC25A22 and SIRT3 had a high positive correlation and that their expression was greater in LUAD tissues than in adjacent tissues. The RSL3-induced ferroptosis of LUAD led to upregulation of SLC25A22 and SIRT3, and SIRT3 protected RSL3-induced LUAD from ferroptosis in vitro and in vivo. At the molecular level, SIRT3 bound with SLC25A22 and deacetylated this protein. Targeting SIRT3 enhanced the acetylation of SLC25A22, decreased its ubiquitination, and promoted 26S proteasome degradation in LUAD cells. Therefore, our results demonstrated that SIRT3 protected LUAD cells from RSL3-induced ferroptosis, and this effect is at least partially due to its deacetylation of SLC25A22, revealing that the SIRT3-SLC25A22 axis has an important role in regulating the ferroptosis of LUAD cells.

## 1. Introduction

Ferroptosis is an iron-dependent form of regulated cell death (RCD) that was first described more than a decade ago [1]. Many cancer cells have high sensitivity to ferroptosis, so targeted upregulation of ferroptosis has promise as a therapeutic strategy [2,3,4]. Although researchers have identified the crucial roles of several subcellular organelles in ferroptosis, the contribution of mitochondria and the underlying mechanisms responsible for ferroptosis of cancer cells are still largely unknown [5]. A 2019 study by Gao et al. found that mitochondria played a key role in the induction of ferroptosis following cysteine deprivation [6].

The solute carrier family 25 (SLC25) consists of 53 proteins that are located in the inner membrane of mitochondria (IMM), which has highly selective permeability. These proteins transport small biological molecules, including nucleotides, amino acids, short-chain fatty acids, short-chain carboxylic acids, and cofactors, across the IMM [7]. Thus, SLC25 proteins have important functions in regulating metabolic processes, such as the tricarboxylic acid (TCA) cycle, oxidative phosphorylation (OXPHOS), and fatty acid oxidation (FAO), and are crucial for maintaining mitochondrial function and homeostasis [7]. Proteins in this family have also emerged as potential targets for various cancer therapies [8,9,10,11,12]. The present study focused on SLC25A22, a mitochondrial glutamate transporter that has roles in the pathogenesis of diverse cancers. For example, this gene promotes tumor progression and inhibits the immunotherapeutic response in colorectal cancers with mutations of the *KRAS* gene [13,14,15]. It maintains the proliferation and survival of colorectal cancer cells with KRAS mutations by promoting the intracellular synthesis of aspartate [13]. In colorectal cancer cells with mutant KRAS, it-mediated glutaminolysis reduces DNA demethylation to increase WNT Signaling, stemness, and drug resistance [14]. In addition, in gallbladder cancer, it promotes the cell proliferation and metastasis by activating the MAPK/ERK pathway [16], and it confers radioresistance to glioblastoma (GBM) by altering glutamate metabolism [17].

Sirtuin 3 (SIRT3, a human homologue of SIR2) is a mitochondrial NAD-dependent deacetylase [18] that functions as an oncogene in some cancers and as a tumor suppressor gene in other cancers [19]. There is evidence that SIRT3 regulates cellular iron metabolism and cancer growth by repressing iron regulatory protein 1 (aconitase) [20]. Other studies showed that SIRT3 promoted ferroptosis in some tumors but inhibited ferroptosis in other tumors [21,22]. We previously demonstrated that targeted knockdown of SIRT3 sensitized glioblastoma to ferroptosis by promoting mitophagy and inhibiting SLC7A11 [23].

Studies of the role of SLC25A22 in PDAC cells showed that this mitochondrial transporter blocked ferroptosis by increasing the levels of glutathione (GSH) and monounsaturated fat acids (MUFAs) in these cells [24]. In the present study, we examined the role of the SIRT3-SLC25A22 axis in protecting lung adenocarcinoma (LUAD) cells from ferroptosis induced by RAS-selective lethal 3 (RSL3).

## 2. Methods

### 2.1. Bioinformatics Analysis

The UALCAN (https://ualcan.path.uab.edu/, accessed on 19 October 2024) online tool was used to analyze the expression of the SLC25A22 and SIRT3 proteins in LUAD tissues and adjacent normal lung tissues, and the correlation of their expression was determined using Spearman’s rank correlation coefficient. The PhosphoSitePlus database (https://www.phosphosite.org/, accessed on 19 October 2024) was used to predict the acetylation site of the SLC25A22 protein.

### 2.2. Reagents and Antibodies

All antibodies and reagents used in this study are listed in Table S1.

### 2.3. Tissue Microarray

Human LUAD tissue microarrays were purchased from Shanghai Xinchao Biotechnology (Shanghai, China) (batch number HLugA150CS04, with attached legitimate ethical documents). Immunohistochemical staining for SLC25A22 and SIRT3 was performed as described below. The correlation between the absence of SIRT3 and SLC25A22 expression in LUAD cells was determined using Spearman’s rank correlation coefficient.

### 2.4. Immunohistochemistry (IHC)

IHC staining was conducted to evaluate the expression of SIRT3 and SLC25A22 in LUAD tumor tissues and paired adjacent normal tissues. First, 4 μm tissue sections were deparaffinized and rehydrated, and antigen retrieval was performed in a pressure cooker by addition of a citric acid buffer for 20 min. Subsequently, the slides were treated with 3% hydrogen peroxide for 10 min to quench endogenous peroxidase activity. The sections were then incubated for 60 min at room temperature with primary antibodies specific for SIRT3 (Cell Signaling Technology, Danvers, MA, USA) or SLC25A22 (Abcam, Cambridge, UK). The sections were then washed with phosphate-buffered saline (PBS), and a biotinylated secondary antibody was added. The immune complexes were visualized using diaminobenzidine (DAB), and the nuclei were counterstained with hematoxylin. All immunostaining results were independently assessed by two pathologists.

### 2.5. Cell Lines and Culture Conditions

Two human LUAD cell lines were used: A549 cells (Cell Bank, Chinese Academy of Sciences, Beijing, China) and H2122 cells (Nanjing Kobai Biotechnology Co., Nanjing, China). Human Embryonic Kidney Cells (HEK293T) were obtained from laboratory stock. The A549 cells were grown in F-12K medium (Sigma, Burlington, MA, USA), the H2122 cells were grown in 1640 medium (Sigma), and the HEK293T cells were grown in DMEM-high glucose medium (Sigma). All cells were cultured with 10% FBS (Gibco, Waltham, MA, USA) and 1% penicillin/streptomycin (Sigma) in a humidified incubator with 5% CO_2_ at 37 °C.

### 2.6. RSL3 Treatment

A549 or H2122 cells were treated with DMSO or RSL3 (4 μM and 8 μM) for 12 h or 24 h at 37 °C in a 5% CO_2_ incubator. Further analyses are described elsewhere in this section.

### 2.7. DFO and Fer-1 Treatment

A549 and H2122 cells were treated with DMSO or deferoxamine (DFO) for 24 h at 37 °C in a 5% CO_2_ incubator. The concentration of DFO used was 100 μM for A549 cells and 5 μM for H2122 cells. A549 and H2122 cells were also treated with DMSO or Ferrostatin-1 (Fer-1) for 24 h at 37 °C in a 5% CO_2_ incubator. The concentration of Fer-1 used was 1 μM for A549 cells and 0.2 μM for H2122 cells. Further analyses are described elsewhere in this section.

### 2.8. Cell Viability Assay

A549 or H2122 cells were seeded in a 96-well plate at a density of 1 × 10^4^ cells per well and incubated at 37 °C with 5% CO_2_ for 12 h. The cells were then pre-treated with DFO or 3-TYP (SIRT3 inhibitor) for 24 h, and then 8 μM RSL3 was added for a further 12 or 24 h. Subsequently, 10 μL of the Cell Counting Kit-8 (CCK8) solution was added to each well, followed by thorough mixing and incubation at 37 °C with 5% CO_2_ for 2 h. Absorbance at 450 nm (A_450nm_) was measured using a microplate reader to determine cell viability.

### 2.9. Western Blotting

Cells were removed from the incubator and washed with 1 × PBS to remove residual medium. Then, sodium dodecyl sulfate (SDS) lysis buffer (50 mM of Tris-HCl [pH 7.4], 150 mM of NaCl, and 2% SDS) was added to the cells. The samples were then heated at 100 °C for 10 min, subjected to 3 sonication cycles (5 s each), and reheated for an additional 10 min to denature the proteins. Protein concentration was measured using a NanoDrop 2000c spectrophotometer (ThermoFisher, Waltham, MA, USA) and standardized to 1 μg/μL by adding 5 × Loading buffer and SDS lysis buffer as needed. Following these steps, the samples were subjected to a final 10 min treatment at 100 °C in preparation for SDS-polyacrylamide gel electrophoresis (SDS-PAGE).

### 2.10. Quantitative Real-Time PCR (RT-qPCR)

Cells in a 6-well plate were washed with 1 × PBS, followed by total RNA extraction using TRNzol Universal Reagent (TIANGEN, Beijing, China). The RNA concentration and purity were determined using a NanoDrop 2000c spectrophotometer. Then, 1 μg of RNA was reverse-transcribed into complementary DNA (cDNA) using the PrimeScript™ RT Master Mix (Takara, Kusatsu, Japan). The cDNA was subsequently used for RT-qPCR with the ChamQ Universal SYBR qPCR Master Mix (Vazyme, Nanjing, China) using primers listed in Table S2.

### 2.11. Construction of the SLC25A22K83R Point Mutation Plasmid

The primers for the SLC25A22K83R point mutation were designed according to the instructions of the Hieff Mut™ Site-Directed Mutagenesis Kit (YEASEN, Shanghai, China), and the detailed sequences are shown in Table S2. Using the pCDNA3.1-SLC25A22-HA plasmid as the template, PCR was performed with the Phanta Max Super-Fidelity DNA Polymerase (Vazyme). The PCR product was then digested with FastDigest DpnI (Thermo Fisher). Subsequently, the digested product was ligated using DNA Ligation Kit Ver.2.1 (Takara). Finally, the ligation product was transformed into competent cells and plated, and single colonies were picked for sequencing to verify the point mutation effect.

### 2.12. shRNA and Stable Cell Lines

The plasmids utilized in this study were PLKO.1-Puro, PLKO.1-shSIRT3-1-Puro, PLKO.1-shSIRT3-3-Puro, PLKO.1-shSIRT3-4-Puro, PGMLV-CMV-MCS-PGK-Blasticidin, PGMLV-CMV-SLC25a22-3×Flag-PGK-Blasticidin, PCDNA3.1, PCDNA3.1-SIRT3-Flag, PCDNA3.1-SLC25A22-HA, and PCDNA3.1-SLC25A22(K83R)-HA. PCDNA3.1-SLC25A22(K83R)-HA was constructed using the Hieff Mut^TM^ Site-Directed Mutagenesis Kit (YEASEN). The H248Y Sirt 3 mutant was constructed as previously reported [25]. HEK293T cells were transfected with shRNA and PSPA × 2 and PMD.2G plasmids, with the Hieff Trans^®^ Liposomal Transfection Reagent (YEASEN) to package lentiviruses for transfection of A549 and H2122 cells. Infected cells were selected with media containing puromycin or blasticidin for a period exceeding 3 days (puromycin) or 7 days (blasticidin) to establish stable cell sublines.

### 2.13. Mitochondrial Acetylation Assay

Mitochondria were isolated from cultured cells using the Mitochondria Isolation Kit (Abcam, ab110171). Cells were digested with trypsin and then resuspended and centrifuged in 1 × PBS at 370× *g* (4 °C, 10 min). The supernatant was discarded, and the pellet was resuspended in NKM buffer, with a buffer volume 10-fold greater than the pellet volume. Then, centrifugation at 370× *g* was performed twice (4 °C, 10 min), with removal of the supernatant. The pellet was resuspended in Homogenization Buffer, with a buffer volume 10-fold greater than the pellet volume, and chilled on ice for 10 min. The cells were then disrupted using a mitochondrial homogenizer to achieve more than 60% breakage. An equal volume of pre-chilled 2 M sucrose buffer was added, followed by gentle mixing. The sample was centrifuged twice at 1200× *g* (4 °C, 10 min), and the supernatant was saved. Then, the sample was centrifuged again at 2000× *g* (4 °C, 10 min); the supernatant was collected and centrifuged at 7000× *g* (4 °C, 10 min), and the resulting supernatant was discarded. The pellet was resuspended in Mitochondrial Suspension Buffer, with a buffer volume 3-fold greater than the pellet volume, centrifuged at 9500× *g* (4 °C, 5 min), and the mitochondria-enriched pellet was collected. This pellet was used for Western blotting and measurements of mitochondrial pan-acetylation.

### 2.14. Lipid Peroxidation (LPO) Assay

A549 or H2122 cells in 6-well plates were treated with DMSO or RSL3 for 12 h or 24 h and then washed twice with 1 × PBS. After incubation with 5 μM of the BODIPY fluorophore (Invitrogen, Waltham, MA, USA) at 37 °C and 5% CO_2_ for 30 min, cells were collected, centrifuged at 800 rpm for 5 min, washed twice more with 1 × PBS, and resuspended in 200 μL of FACS buffer. Lipid peroxidation was detected by flow cytometry (excitation: 665 nm, emission: 676 nm).

### 2.15. Mitochondrial Fe^2+^

A549 cells were plated in 3.5 cm glass-bottom dishes for 12 h and were then treated with 8 μM RSL3 for an additional 12 h. The medium was then aspirated, and the cells were washed 3 times with 1 mL of serum-free F12 medium. The sample was then incubated with 5 μmol/L of the Mito-FerroGreen fluorophore (Dojindo, Kumamoto, Japan) in serum-free F12 medium for 30 min at 37 °C with 5% CO_2_. Then, the supernatant was removed, the cells were washed 3 times with serum-free F12 medium, and the cells were then examined using a Leica TCS Sp8 STED Confocal Super-Resolution Microscope (Nikon A1R, Tokyo, Japan).

### 2.16. Mitochondrial ROS

A549 cells were cultured in 6-well plates for 12 h and then treated with 8 μM RSL3 for an additional 12 h. The medium was aspirated, and the cells were washed twice with 1 mL of 1 × PBS and then incubated in the dark with 5 μM of the MitoSOX fluorophore (Invitrogen) working solution in 1 × PBS for 30 min at 37 °C with 5% CO_2_. After incubation, the cells were collected, centrifuged at 800 rpm for 5 min, and washed twice with 1 × PBS. Finally, the cells were resuspended in 200 μL of FACS buffer and analyzed using a CytoFlex S flow cytometer (Beckman Coulter, Brea, CA, USA), with detection through the red fluorescence channel (MitoSOX, Invitrogen, Carlsbad, CA, USA).

### 2.17. Transmission Electron Microscopy

A549 cells were plated in 10 cm dishes for 12 h and then treated with 8 μM RSL3 for an additional 12 h. The cells were carefully digested, and digestion was terminated promptly with serum-containing medium. The cells were then centrifuged at 800 rpm for 5 min, resuspended in pre-chilled 1 × PBS, transferred to a 1.5 mL Eppendorf tube, and centrifuged again at 800 rpm for 5 min. The supernatant was discarded, 1.2 mL of fixing solution (2.5% glutaraldehyde in 1 × PBS) was added, the solution was gently mixed via pipetting, and the cells were maintained overnight at 4 °C. Subsequently, the resulting cell pellets were fixed in 2% osmium tetroxide, dehydrated using a graded series of ethanol and propylene oxide, and embedded in Epon and stored at 60 °C for 20 h. Ultrathin sections were placed onto 200-mesh copper grids, double-stained with lead citrate and uranyl acetate, and examined using transmission electron microscopy.

### 2.18. Animal Experiments

All animal experiments were approved by the Animal Care Committee of the School of Medicine, Shanghai Jiao Tong University (Shanghai, China) (the ethical approval code is RA-2024-197, and approval date is 21 February 2024) and were performed following the Guidelines of the Care and Use of Laboratory Animals issued by the Chinese Council on Animal Research. In brief, 6-week-old nude male BALB/c nu/nu athymic nude mice were acclimated to the laboratory environment for 1 week and then randomly divided into 4 groups (5 mice per group). The mice were given subcutaneous injections of A549 cells in 200 µL of 1 × PBS (5 × 10^6^ cells per injection). One week later, calipers were used to measure tumor size every 3 days. Once the tumor volume (0.5 × L × W^2^) reached 100 mm^3^, 3-TYP (50 mg/kg) and/or RSL3 (50 mg/kg) were injected into the tumors every three days. One week later, the mice were sacrificed, and tumor weights were measured.

### 2.19. Co-Immunoprecipitation (Co-IP)

For the acetylation IP assay, total mitochondria were extracted from A549 or HEK293T cells, as described above. Then, 500 μL of IP buffer (150 mM of NaCl, 50 mM of Tris-base [pH 7.8], 0.25% deoxycholic acid, and 0.3% NP40) with a protease inhibitor cocktail (PIC) and 1 μM of nicotinamide (NAM) were added. The samples were lysed on a shaker at 4 °C for 30 min, centrifuged at 12,000 rpm (4 °C, 5 min), and the supernatant was collected for protein quantification using the BCA assay. Equal amounts of input and total protein were collected, as well as anti-GC-1 antibody (Abcam) or M2-Flag affinity gel (Sigma) or Anti-HA Magnetic Beads (MCE, Monmouth Junction, NJ, USA), followed by incubation overnight at 4 °C. When using the anti-GC-1 antibody, 20 μL of equilibrated Protein A Magnetic Beads (Smart-Lifesciences, Changzhou, China) was included. Finally, the beads were washed 3 times in the IP buffer, the supernatant was discarded, and 50 μL of 1 × loading buffer was added for Western blot analysis.

For ubiquitination IP assays and IP experiments involving SLC25A22 and SIRT3, 500 μL of IP buffer (150 mM of NaCl, 50 mM of Tris [pH 7.4], 0.5% deoxycholic acid, 1% NP40, 0.1% SDS) with PIC was added to the A549 or HEK293T cells. The cells were lysed on a shaker at 4 °C for 20 min, subjected to 3 sonication cycles (4 s each), and then returned to the shaker for an additional 10 min. Then, the sample was centrifuged at 12,000 rpm (4 °C, 5 min), and the pellet was discarded. All subsequent steps were described above.

### 2.20. GSH

For preparation of tumor tissue samples, 10 mg of tissue was added to 100 μL of protein removal reagent M solution with 2 grinding balls and processed using an automatic cryogenic grinder (Shanghai Jingxin, Shanghai, China) at −20 °C and 65 Hz for 120 s. Afterward, the samples were stored at 4 °C for 10 min, centrifuged at 10,000× *g* (4 °C, 10 min), and the supernatant was collected for determination of GSH. The preparation of cell samples and subsequent detection of GSH were performed using the GSH and GSSG Assay Kit (Beyotime, Haimen, China, S0053).

### 2.21. Statistical Analysis

All statistical analyses were conducted using SPSS version 26.0 and GraphPad Prism version 9. Continuous data are presented as means ± standard deviations (SDs). Differences between experimental groups were analyzed using one-way analysis of variance (ANOVA) or Student’s *t*-test. A difference with a *p*-value less than 0.05 was considered statistically significant.

## 3. Results

### 3.1. High Expression of SLC25A22 in LUAD Predicts Poor Prognosis

**Figure 1:**
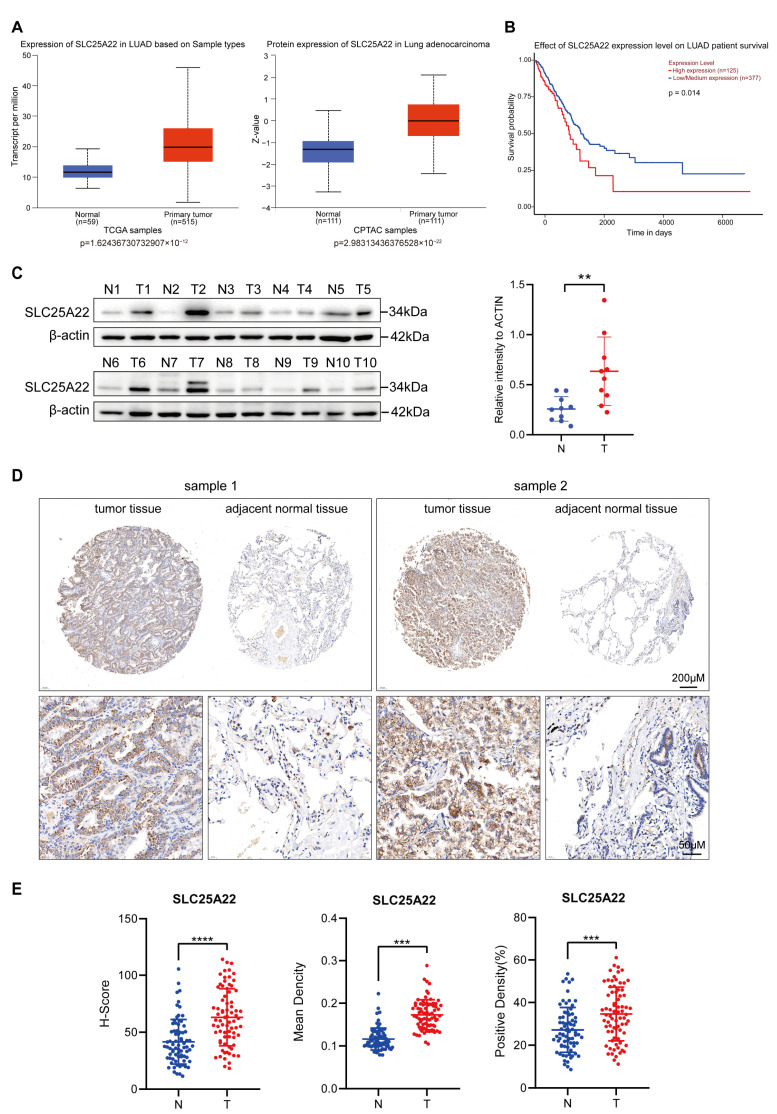
High expression of SLC25A22 in LUAD predicts poor prognosis. (**A**) UALCAN online analysis revealed higher expression of *SLC25A22* mRNA and protein in LUAD tissues than in normal lung tissues. (**B**) UALCAN online analysis revealed that higher expression of SLC25A22 in LUAD predicted poorer prognosis. (**C**) Immunoblotting revealed higher expression of SLC25A22 in tumor tissues than in paired adjacent tissues of 10 patients with LUAD. ** *p* < 0.01. (**D**) Representative results of a tissue microarray staining assay showed that SLC25A22 expression was higher in tumor tissues than paired adjacent tissues of patients with LUAD. (**E**) Quantitative results for SLC25A22 expression in the tissue microarray assay. *** *p* < 0.001, **** *p* < 0.0001.

To examine the possible role of SLC25A22 in LUAD, we first analyzed the expression of SLC2522 protein and mRNA in LUAD tissue and adjacent normal tissue from two databases: The Cancer Genome Atlas (TCGA) and Clinical Proteomic Tumor Analysis Consortium (CPTAC). The results showed that the levels of the SLC25A22 protein and mRNA were higher in LUAD tissues than in normal tissues (Figure 1A). In addition, UALCAN online analysis showed that high expression of *SLC25A22* predicted poor patient prognosis (Figure 1B). We also collected 10 pairs of tumors and adjacent tissues of LUAD patients from Shanghai Zhongshan Hospital, Affiliated with Fudan University. Western blotting showed higher expression of SLC25A22 in LUAD tissues than in paired normal tissues (Figure 1C). Moreover, a tissue microarray staining assay also showed higher expression of the SLC25A22 protein in LUAD tissues than in paired normal tissues (Figure 1D,E and Figure S1). Collectively, these data demonstrate that SLC25A22 had high expression in LUAD tissues and that high expression of SLC25A22 predicted poor patient prognosis.

### 3.2. SIRT3 Expression Is Positively Correlated with SLC25A22 Expression in LUAD

**Figure 2:**
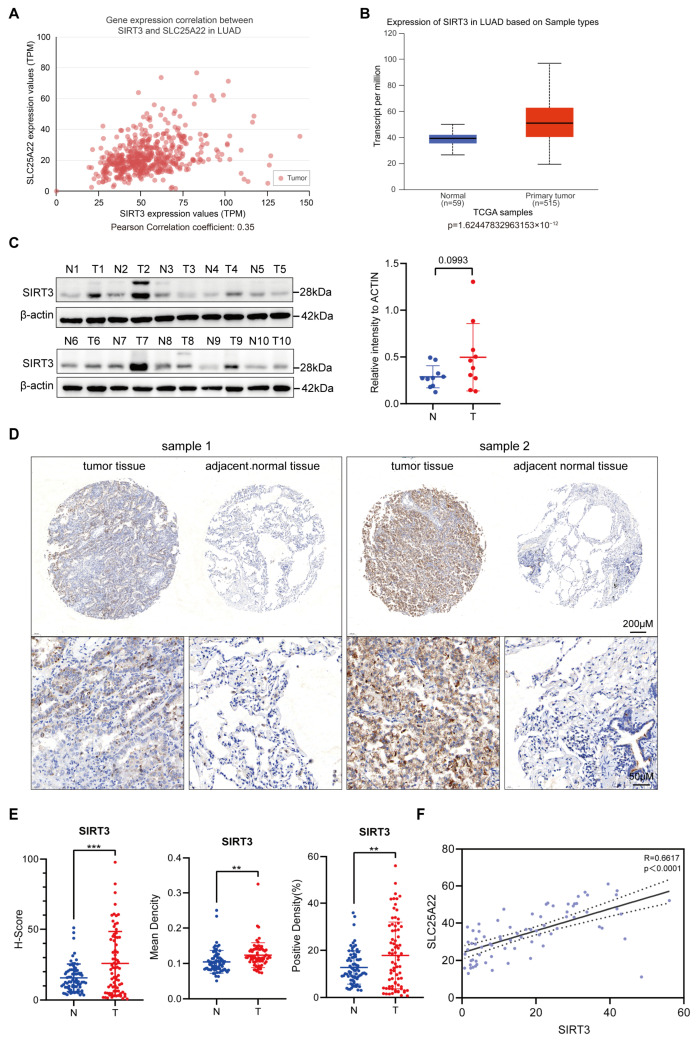
SIRT3 has high expression and is positively correlated with SLC25A22 expression in LUAD, with a Pearson correlation coefficient of 0.35. (**A**) UALCAN online analysis revealed that SIRT3 expression was significantly correlated with SLC25A22 expression in LUAD tissues. (**B**) UALCAN online analysis revealed higher expression of SIRT3 in LUAD tissues than in normal lung tissues. (**C**) Immunoblotting revealed higher expression of SIRT3 in tumor tissues than in paired adjacent normal tissues from LUAD patients (*n* = 10). (**D**) Representative results of the tissue microarray staining assay showed that SIRT3 expression was higher in tumor tissues than in paired adjacent tissues of patients with LUAD. (**E**) Quantitative results for SIRT3 expression in the tissue microarray assay. ** *p* < 0.01, *** *p* < 0.001. (**F**) Correlation analysis revealed that SIRT3 expression was highly correlated with SLSC25A22 expression in LUAD tissues, with a Pearson correlation coefficient of 0.6617.

Previous research identified SLC25A22 as a key mitochondrial transporter that inhibited ferroptosis in various cancers [26]. Because SIRT3 (a mitochondrial deacetylase) plays critical roles in the regulation of ferroptosis in other cancers, such as GBM, we performed bioinformatic analysis using UALCAN to examine the relationship of the expression of SIRT3 and SLC25A22. The results show a significant positive correlation in the expression of these two proteins (Pearson’s r: 0.35; Figure 2A). In agreement, TCGA analysis, Western blotting of 10 paired LUAD tissue samples, and tissue microarray staining identified higher expression of the SLC25A22 protein in LUAD tissues than in paired normal tissues (Figure 2B–E and Figure S2). In addition, the staining results of a tissue microarray confirmed the high positive correlation in the expression of these two proteins (Figure 2F).

### 3.3. SLC25A22 and SIRT3 Are Both Upregulated During RSL3-Induced Ferroptosis of LUAD Cells

**Figure 3:**
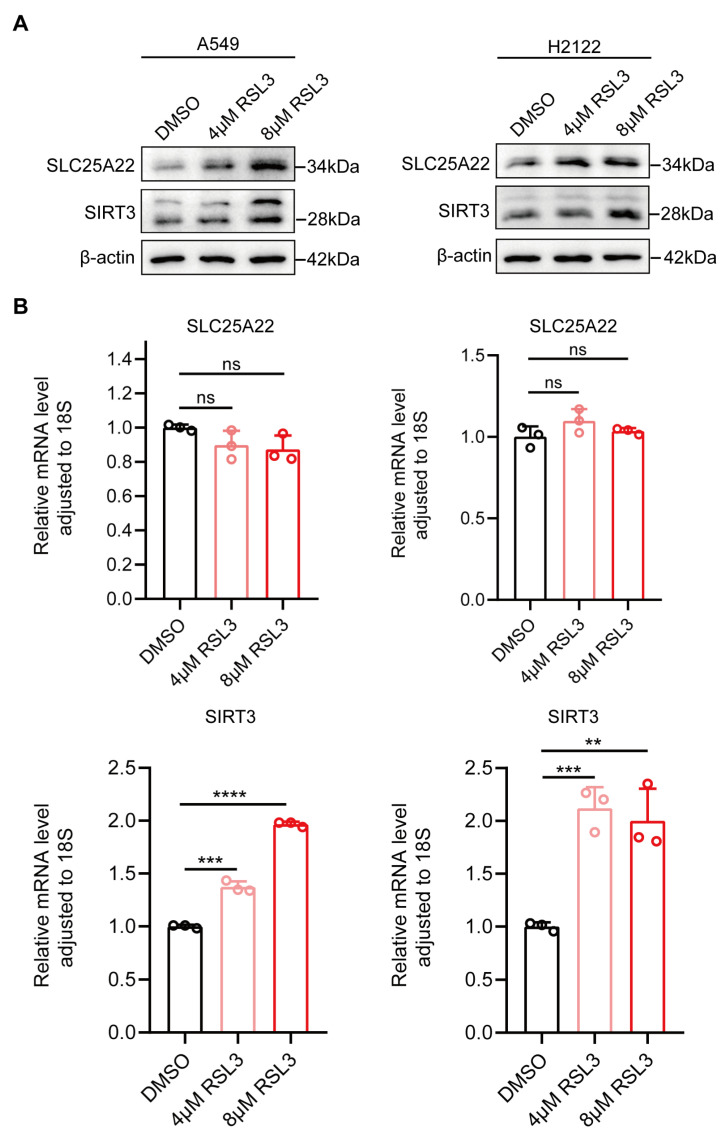
SLC25A22 and SIRT3 proteins are upregulated during RSL3-induced ferroptosis of LUAD cells. (**A**) RSL3 increased the expression of SLC25A22 and SIRT3 proteins in LUAD cells. (**B**) RSL3 increased the expression of *SIRT3* mRNA but not *SLC25A22* mRNA in LUAD cells. ** *p* < 0.01, *** *p* < 0.001, **** *p* < 0.0001, ns: no significance.

We then investigated the biological roles of SLC25A22 and SIRT3 in the ferroptosis of LUAD cells by treating two LUAD cell lines (A549 and H2122) with the ferroptosis inducer RSL3. As shown in our previous work [27], analysis of cell viability showed that RSL3 induced the death of A549 and H2122 cells in a dose-dependent manner, which can be partially rescued by ferroptosis inhibitors DFO and Fer-1 (Figure S3A). In addition, RSL3 increased the expression of two ferroptosis-related proteins (FRPs, heme oxygenase-1 [HO-1] and ferritin), decreased the expression of glutathione peroxidase 4 (GPX4, a ferroptosis suppressor) (Figure S3B), and increased the level of cellular lipid peroxide (LPO) in LUAD cells in a dose-dependent manner (Figure S3C). These data indicate that treatment of LUAD cells with RSL3 (4 μM or 8 μM) increased the ferroptosis of these cells and also increased the expression of the SLC25A22 and SIRT3 proteins (Figure 3). However, RSL3 only increased the expression of the SLC25A22 protein, but it increased the expression of the SIRT3 mRNA and protein. Moreover, erastin, another kind of ferroptosis inducer by inhibiting cystine uptake, was used to induce LUAD cell ferroptosis. And upregulation of SLC25A22 and SIRT3 was observed (Figure S4). More importantly, we showed that in Figure S5, when SIRT3-knockdown, SLC25A22 upregulation in A549 cells in response to RSL3 treatment was strikingly inhibited, revealing that SLC25A22 upregulation induced by RSL3 is dependent on SIRT3 during ferroptosis.

### 3.4. SIRT3 Protects LUAD Cells from RSL3-Induced Ferroptosis In Vitro

**Figure 4:**
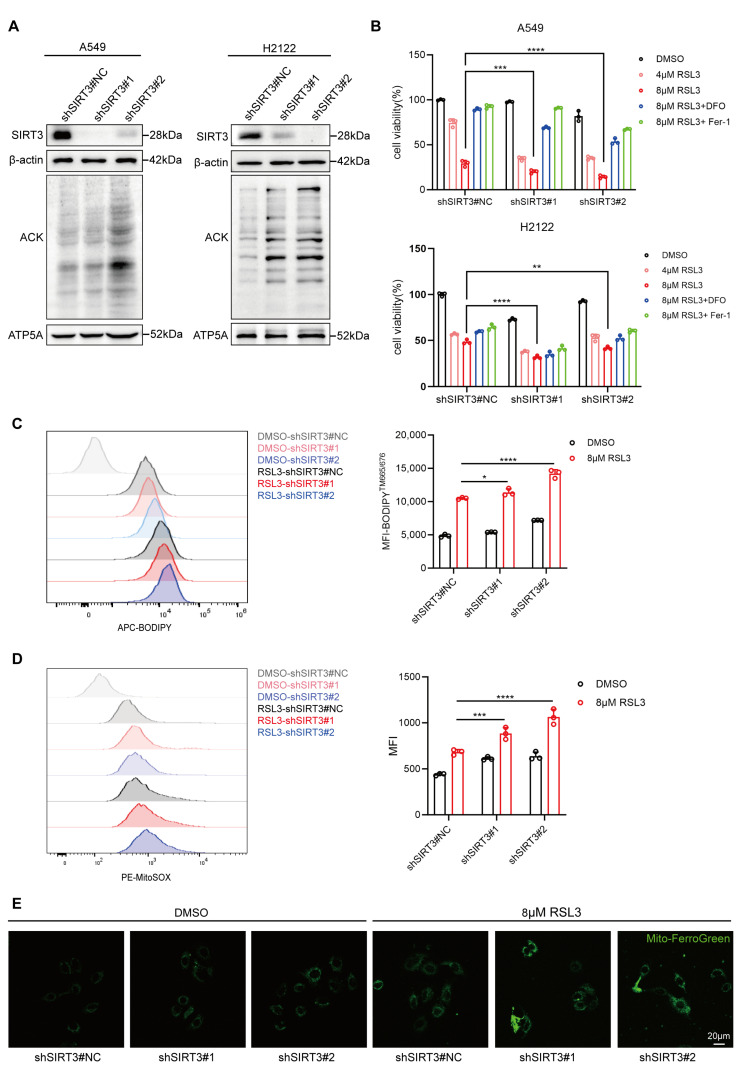
SIRT3 protects LUAD cells from RSL3-induced ferroptosis in vitro. (**A**) Western blot analyses of SIRT3 and mitochondrial pan-acetyllysine (AC-LYS) levels in LUAD cells transfected with SIRT3 knockdown lentiviruses or a control lentivirus. (**B**) A549 and H2122 cells transfected with shNC, shSIRT3#1, and shSIRT3#2 were treated with DMSO (control), RSL3 (4 μM and 8 μM), or RSL3 (8 μM) combined with deferoxamine (DFO) or Ferrostatin-1 (Fer-1) for 24 h. Cell viability was assessed using the CCK-8 assay. The concentration of DFO used was 100 μM for A549 cells and 5 μM for H2122 cells; The concentration of Fer-1 used was 1 μM for A549 cells and 0.2 μM for H2122 cells. (**C**–**E**) A549 cells transfected with shNC, shSIRT3#1, and shSIRT3#2 were treated with DMSO or 8 μM RSL3 for 12 h. (**C**) Inhibition of SIRT3 expression promoted accumulation of LPO in A549 cells. Intracellular lipid peroxidation levels were measured via flow cytometry using the BODIPY™ 665/676 probe. (**D**) Inhibition of SIRT3 expression promoted accumulation of mitochondrial ROS in A549 cells. Mitochondrial ROS levels were measured via flow cytometry using MitoSOX Red. (**E**) Inhibition of SIRT3 expression promoted accumulation of mitochondrial Fe^2+^ in A549 cells. Mitochondrial Fe^2^⁺ levels were visualized via confocal microscopy using Mito-FerroGreen. * *p* < 0.05, ** *p* < 0.01, *** *p* < 0.001, **** *p* < 0.0001.

Because SIRT3 is highly expressed in LUAD tissues and during RSL3-induced ferroptosis of LUAD cells, we hypothesized that this protein might protect LUAD cells from RSL3-induced ferroptosis. We therefore established two LUAD cell lines (A549 and H2122) that were transfected with a lentivirus that interfered with SIRT3. Western blotting showed that the mitochondrial proteins in these cells had significantly increased levels of lysine acetylation (Figure 4A). Then, we treated cells with SIRT3 interference and negative control (NC) cells with RSL3 (4 μM or 8 μM), with or without DFO or Fer-1. The results show that SIRT3 knockdown decreased the viability of both lines of LUAD cells and that DFO or Fer-1 treatment led to partial rescue (Figure 4B).

To further examine the role of SIRT3 in antagonizing ferroptosis in LUAD, we measured LPO production in A549 cells before and after SIRT3 knockdown. The results show that SIRT3 knockdown increased the level of LPO upon treatment with 8 μM of RSL3 (Figure 4C). In addition, inhibition of SIRT3 expression strikingly increased the mitochondrial levels of ROS and Fe^2+^ (Figure 4D,E). All these data demonstrate that SIRT3 (a mitochondrial deacetylase) protected LUAD cells from RSL3-induced ferroptosis. We also found that 3-TYP (a specific inhibitor of SIRT3) obviously reduced the viability and increased the LPO level in A549 cells that were treated with RSL3 (Figure S6). In other words, the antagonistic effect of SIRT3 on the ferroptosis of LUAD cells depends on its deacetylase activity.

### 3.5. SIRT3 Protects LUAD from RSL3-Induced Ferroptosis In Vivo

**Figure 5:**
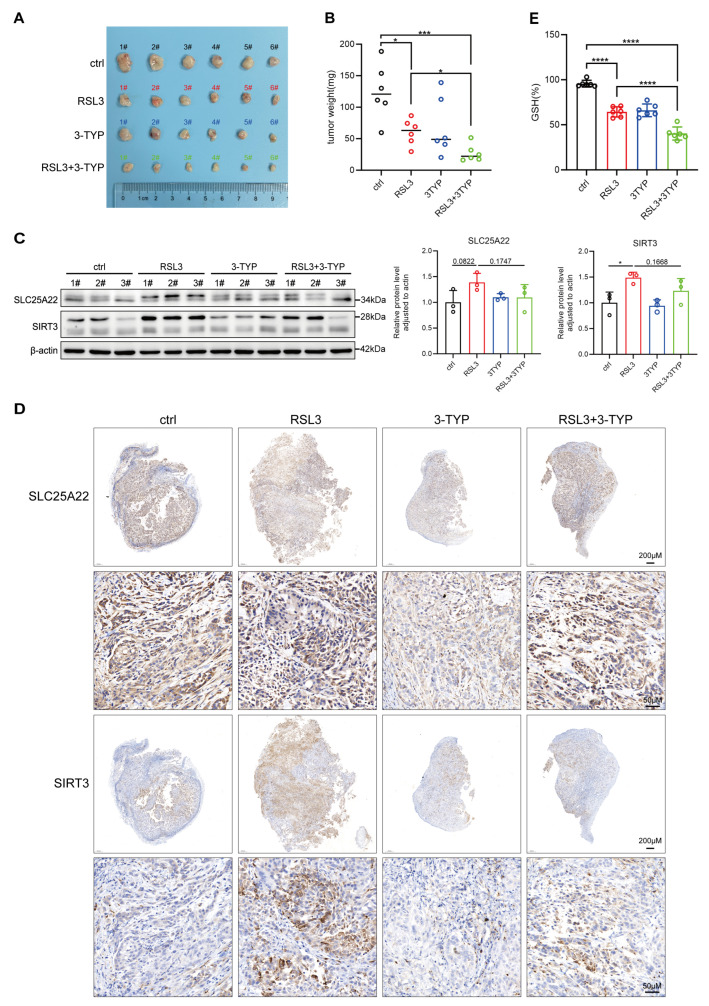
SIRT3 protects LUAD from RSL3-induced ferroptosis in vivo. (**A**) After a week of acclimation, 6-week-old immunodeficient nude mice were randomly divided into four groups (six mice per group). Tumor models were established by subcutaneously injecting A549 cells (5 × 10^6^ cells per mouse). When the tumor volume reached 100 mm^3^, intratumoral injections were administered as follows: DMSO, RSL3 (40 mg/kg), 3-TYP (40 mg/kg), or a combination of RSL3 (40 mg/kg) and 3-TYP (40 mg/kg). The injections were performed every 3 days for a total of three treatments. After the completion of treatment, the mice were sacrificed, and the tumors were excised, weighed, and photographed. 3-TYP + RSL3 significantly impeded the growth of LUAD in xenograft mice compared to the control and RSL3 groups. (**B**) Quantitative analysis of tumor weight revealed significant differences among the control, RSL3, 3-TYP, and RSL3 + 3-TYP treatment groups. (**C**) Western blotting was used to detect the expression of SLC25A22 and SIRT3 proteins in tumor tissues from the DMSO control group, RSL3-treated group, 3-TYP-treated group, and the combinatory treatment group (left). Relative quantification of band intensities was also performed (right). (**D**) Immunohistochemistry assay was used to evaluate the expression of SLC25A22 and SIRT3 proteins in tumor tissues from the DMSO control group, RSL3-treated group, 3-TYP-treated group, and the combinatory treatment group. (**E**) GSH levels were measured in tumor tissues from the DMSO control group, RSL3-treated group, 3-TYP-treated group, and the combination treatment group. * *p* < 0.05, *** *p* < 0.001, **** *p* < 0.0001.

We then performed studies with a xenograft mouse model to confirm the antagonistic effect of SIRT3 on RSL3-induced ferroptosis of LUAD in vivo. The results show that intratumoral injection of RSL3 significantly decreased tumor growth and, more interestingly, that inhibition of SIRT3 by 3-TYP further attenuated the growth of the implanted tumors (Figure 5A). These results confirm the effect of SIRT3 on ferroptosis and tumor growth in vivo. RSL3 alone and 3-TYP alone decreased the level of GSH in tumors, and both agents together led to a greater decrease (Figure 5B). Furthermore, inhibition of SIRT3 activity by 3-TYP tended to decrease the expression of SLC25A22, although this effect was not statistically significant (Figure 5C,D). In addition, we found that the GSH level was significantly lower in the RSL3 group than in the control, and 3-TYP treatment further decreased the GSH level in tumors, indicating that the inhibition of SIRT3 promotes LUAD cell ferroptosis in vivo (Figure 5E).

### 3.6. SIRT3 Binds to and Deacetylates SLC25A22

**Figure 6:**
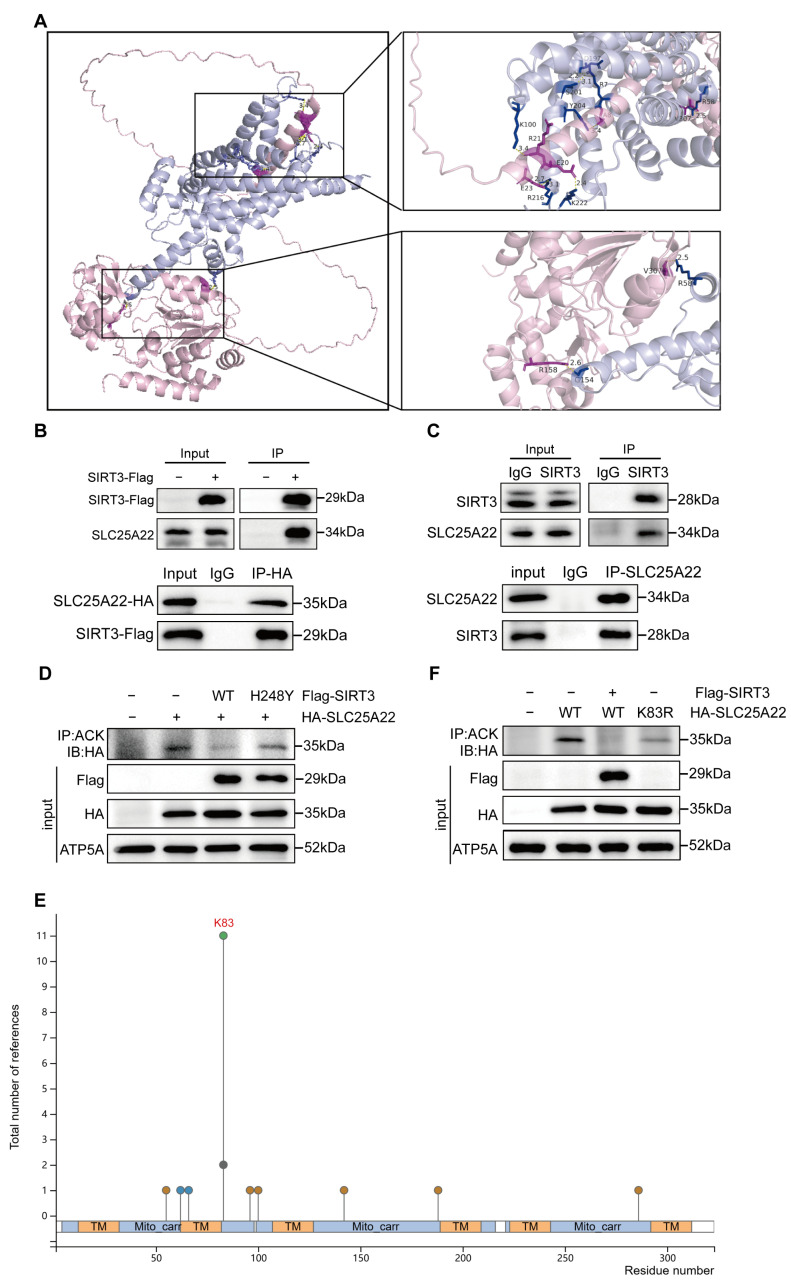
SLC25A22 binds with and deacetylates SIRT3. (**A**) AlphaFold3 (www.alphafold.ebi.ac.uk, accessed on 18 September 2024) predicted the interaction between SLC25A22 (UniProtKB: Q9H936, shown in blue) and SIRT3 (UniProtKB: Q9NTG7, shown in pink). The figure highlights the residues involved in interactions within 5 Å (deep pink for SIRT3 and deep blue for SLC25A22) and hydrogen bonds (represented by yellow dashed lines), with hydrogen bond lengths annotated. (**B**) HEK293T cells were transfected with Flag-SIRT3 and HA-SLC25A22. Western blot analysis of SLC25A22 levels following immunoprecipitation with anti-Flag antibody (upper panel) or anti-HA antibody (lower panel). (**C**) Western blot analysis of SLC25A22 levels following immunoprecipitation with anti-SIRT3 antibody (upper panel) or anti-SLC25A22 antibody (lower panel) in A549 cells. (**D**) HEK293T cells were transfected with HA-SLC25A22 and either Flag-SIRT3WT or Flag-SIRT3H248Y (Histidine at position 248 mutated to tyrosine). Western blot analysis of HA-SLC25A22 levels following immunoprecipitation with Acetylated-Lysine(ACK) Antibody. (**E**) PhosphoSitePlus online analysis (https://www.phosphosite.org/, accessed on 18 September 2024) predicted that lysine 83 (K83) of SLC25A22 was the acetylation site. (**F**) HEK293T cells were transfected with Flag-SIRT3 and/or HA-SLC25A22WT, HA-SLC25A22K83R (Lysine at position 83 mutated to arginine). Western blot analysis of HA-SLC25A22 levels following immunoprecipitation with Acetylated-Lysine(ACK) Antibody.

To illuminate the possible molecular relationship of SLC25A22 and SIRT3 in the ferroptosis of LUAD cells, we first used AlphaFold3 to predict the binding of SIRT3 and SLC25A22 (Figure 6A). In agreement with the AlphaFold3 results, the results of exogenous and endogenous Co-IP assays confirmed a strong binding between SIRT3 and SLC25A22 in 293T and A549 cells (Figure 6B,C). These results indicated that mitochondrial SLC25A22 might be the potential substrate of SIRT3 in the mitochondria. Indeed, the results of the IP assay show that SIRT3 deacetylated SLC25A22, but the H248Y Sirt 3 mutant could not (Figure 6D). PhosphoSitePlus online analysis (https://www.phosphosite.org/, accessed on 19 October 2024) predicted that lysine 83 (K83) of SLC25A22 was the potential acetylation site (Figure 6E). We therefore mutated K83 into arginine (R) and found that, using IP assay, the K83R mutation dramatically decreased the SLC25A22 acetylation level, indicating that K83 is indeed the major acetylation site of SLC25A22 (Figure 6F).

### 3.7. SIRT3 Decreases Ubiquitination and Increases Stability of SLC25A22

**Figure 7:**
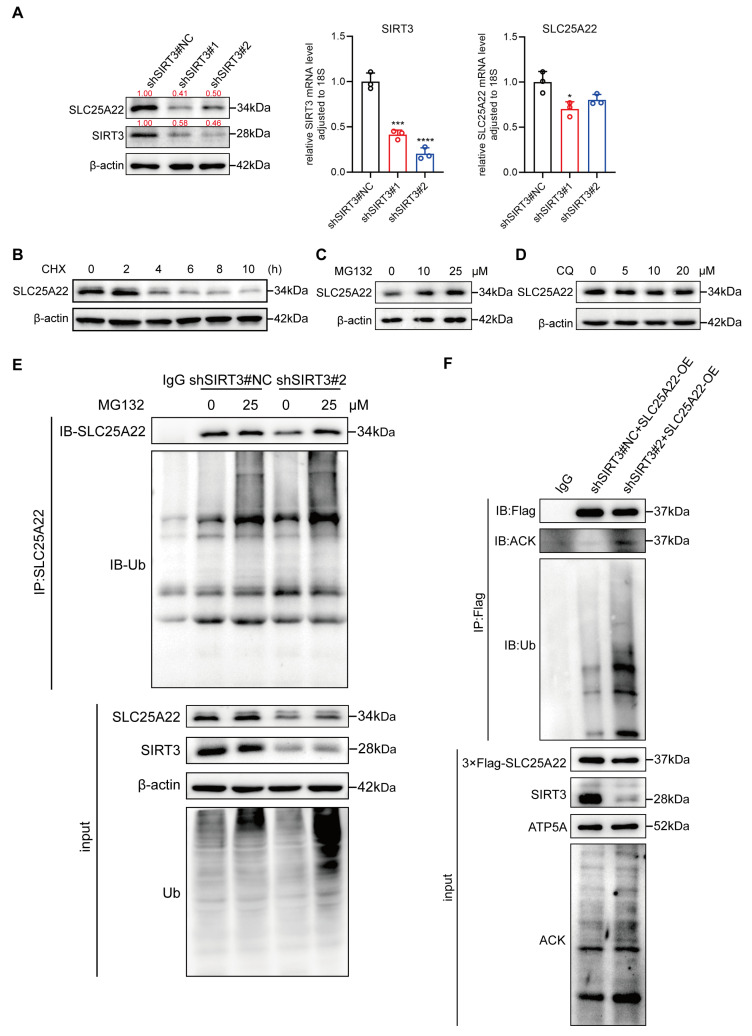
SIRT3 decreases the ubiquitination and deacetylates and stabilizes the SLC25A22 protein. (**A**) Total protein and RNA were extracted from A549 cells transfected with shNC, shSIRT3#1, and shSIRT3#2. The expression levels of SLC25A22 protein and mRNA were detected using Western blot and RT-qPCR, respectively. (**B**–**D**) A549 cells were treated with 100 μg/mL of cycloheximide (CHX) (**B**) proteasome inhibitor MG132 (**C**) or lysosome inhibitor chloroquine (CQ) (**D**) for the indicated times. SLC25A22 protein levels were analyzed using Western blot. (**E**) A549 cells stably transfected with shNC and shSIRT3#2 were treated with DMSO or 25 μM MG132 for 8 h. Total cell lysates were subjected to co-immunoprecipitation using anti-SLC25A22 antibody, followed by Western blot analysis to detect ubiquitination levels. (**F**) A549 cells stably transfected with shNC and shSIRT3#2 overexpressing SLC25A22-3×Flag were used for co-immunoprecipitation with anti-Flag antibody. Acetylated-Lysine (ACK) levels of SLC25A22 were detected using Western blot. * *p* < 0.05, *** *p* < 0.001, **** *p* < 0.0001.

Our further studies of LUAD cells showed that the inhibition of SIRT3 decreased the expression of the SLC25A22 protein more significantly than at the mRNA level (Figure 7A), indicating that SIRT3 altered SLC25A22 expression at the post-transcriptional level. Cycloheximide (CHX) treatment showed that SLC25A22 was a short-lived protein with a half-life of about 4 h (Figure 7B). To examine the mechanism by which SLC25A22 affected protein stability, we treated A549 cells with a 26S-proteasome inhibitor (MG132) or a lysosome inhibitor (chloroquine, CQ). The results show that MG132 increased the level of the SLC25A22 protein in a dose-dependent manner (Figure 7C,D), suggesting that the SLC25A22 protein might be regulated by the ubiquitin–proteasome system.

We also investigated the regulatory role of SIRT3 on the stability of the SLC25A22 protein by adding MG132 to A549 cells that had SIRT3 knockdown and NC A549 cells. An IP assay showed that SIRT3 knockdown decreased the expression and increased the ubiquitination of SLC25A22, and MG132 partially reversed SIRT3-mediated downregulation of SLC25A22 (Figure 7E). These results confirmed that the SIRT3-mediated regulation of the stability of the SLC25A22 protein depended on the ubiquitin–proteasome system. In addition, SIRT3 knockdown increased SLC25A22 acetylation and decreased the level of the SLC25A22 protein due to its increased ubiquitination (Figure 7F). Collectively, these data demonstrate that SIRT3 reduces the ubiquitination of SLC25A22 and stabilizes the SLC25A22 protein via deacetylation.

### 3.8. SIRT3 Protects LUAD Cells from RSL3-Induced Ferroptosis at Least Partially Due to Deacetylation of SLC25A22

**Figure 8:**
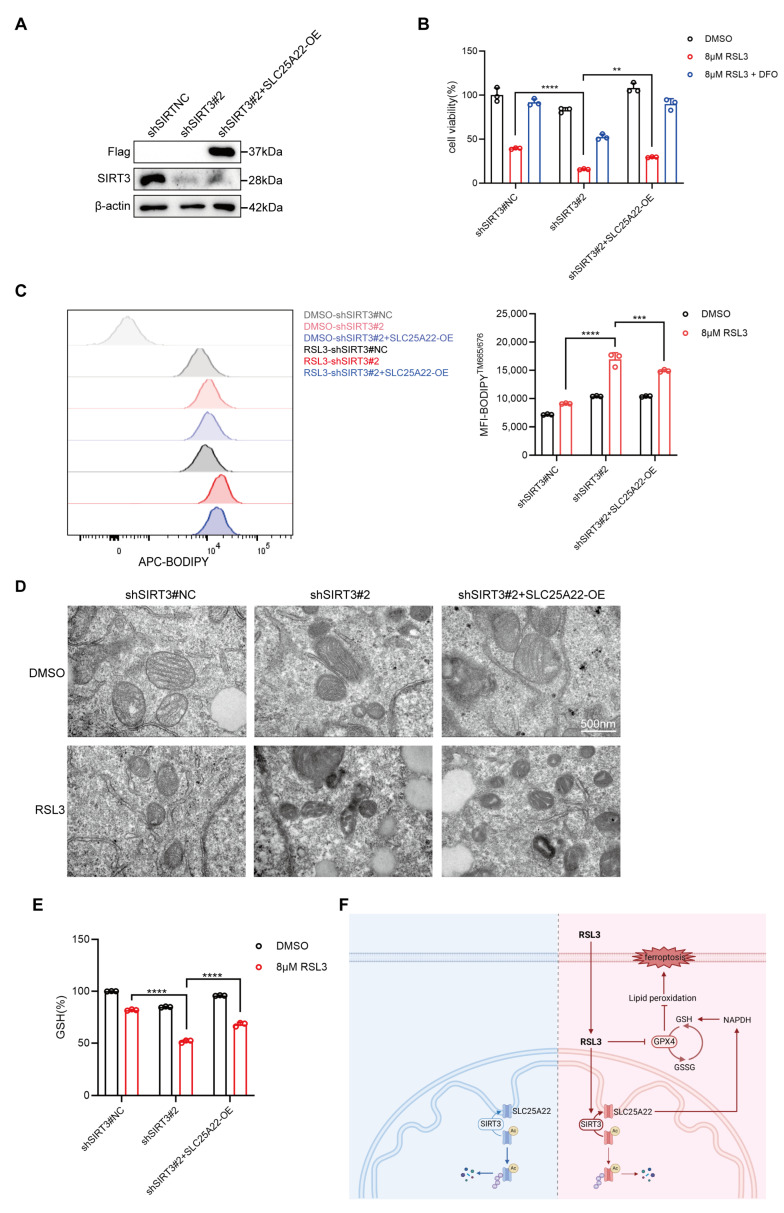
SIRT3 prevents RSL3-induiced ferroptosis of LUAD cells at least partially through SLC25A22. (**A**) Western blotting demonstrated the forced expression of SLC25A22 in A549 cells with SIRT3 knockdown. (**B**) Forced expression of SLC25A22 in A549 cells with SIRT3 knockdown led to partial recovery of cell viability. Cell viability was assessed using the CCK-8 assay. (**C**) Forced expression of SLC25A22 in A549 cells with SIRT3 knockdown led to partial recovery of LPO accumulation following RSL3 treatment. LPO levels were measured using the BODIPY™ 665/676 probe. (**D**) Forced expression of SLC25A22 in A549 cells with SIRT3 knockdown led to partial recovery of mitochondrial morphological changes. Mitochondrial morphological changes were observed using TEM (transmission electronic microscope). (**E**) A549 cells stably transfected with shNC, shSIRT3#2, and shSIRT3#2 + SLC25A22-OE were treated with DMSO, 8 μM of RSL3, or 8 μM of RSL3 + 100 μM of DFO for 12 h. Intracellular GSH levels were measured. Data are presented as mean ± SD. ** *p* < 0.01, *** *p* < 0.001, **** *p* < 0.0001. (**F**) Working model showing that SIRT3 protects LUAD cells from RSL3-induced ferroptosis by deacetylating and stabilizing the mitochondrial glutamate transporter SLC25A22.

To further elucidate the mechanism by which SIRT3 protects LUAD cells from RSL3-induced ferroptosis, we increased the expression of SLC25A22 in A549 cells that had SIRT3 interference (Figure 8A). The results showed that forced expression of SLC25A22 partially rescued cell death in A549 cells that had SIRT3 knockdown and were treated with RSL3 (Figure 8B). LPO detection and transmission electron microscopy showed that SLC25A22 overexpression partially reduced the LPO level and alleviated the mitochondrial ultrastructural damage (Figure 8C,D) that was due to SIRT3 knockdown and RSL3 treatment. In addition, the cellular GSH level (which was decreased by SIRT3 knockdown upon RSL3 treatment) partially recovered after the forced expression of SLC25A22. These data, therefore, indicate that SIRT3 protects LUAD cells from RSL3-induced ferroptosis, at least partially due to its deacetylation of the mitochondrial glutamate transporter and stabilizing SLC25A22 (Figure 8E).

## 4. Discussion

The present study analyzed the possible role of the SIRT3-SLC25A22 axis in the RSL3-induced ferroptosis of LUAD cells. Although previous research showed that SIRT3 and SLC25A22 had important roles in regulating ferroptosis in cancer cells [23,24], the present study is the first to identify a correlation and interaction of these two proteins in antagonizing the RSL3-induced ferroptosis of LUAD cells. In mitochondria, SIRT3 binds and deacetylates SLC25A22, and this decreases its ubiquitination and increases its lifetime. Therefore, the knockdown of SIRT3 leads to the downregulation of SLC25A22 and sensitizes LUAD cells to RLS3-induced ferroptosis in vitro and in vivo. Enhancing SLC25A22 expression in LUAD cells with SIRT3 interference partially rescued cells from RSL3-induced ferroptosis (Figure 8). Hence, targeting the SIRT3-SLC25A22 axis by inducing ferroptosis has potential as a general strategy for the treatment of LUAD.

SIRT3 is reported to be critical for p53-mediated ferroptosis upon ROS-induced stress [22]. To exclude the influence of p53 in our research, we used A549 and H2122 cells to perform the experiments, which is p53 wt and contains p53 Q16L and C176F mutations individually. We observed the same results from the two LUAD cell lines, which indicates that the p53 status is not essential in our research system. SIRT3 is the main deacetylase in mitochondria, and it has many substrates, including mitochondrial isocitrate dehydrogenase 2 (IDH2), long-chain acyl coenzyme A dehydrogenase (LCAD), manganese superoxide dismutase (MnSOD), mitochondrial 3-hydroxy-3-methylglutaryl CoA synthase 2 (HMGCS2), and ornithine transcarbamoylase (OTC, in the urea cycle) [25,26,27,28,29,30]. SIRT3 is critical for maintaining mitochondrial function because it reduces the level of mitochondrial ROS, increases FAO, promotes the urea cycle, and regulates the production of ketone bodies [25,26,27,28,29,30]. We identified SLC25A22, an important IMM transporter, as the novel substrate of SIRT3, thus broadening the biological function of SIRT3. Previous studies identified other proteins in the SLC25 family that regulate ferroptosis, such as SLC25A10, SLC25A11, and SLC25A28 [31,32,33]. However, SLC25A22 is the first protein in this family to be identified as an acetylation substrate during ferroptosis, which promotes the production of GSH and MUFAs. Previous studies reported that SLC25A22 suppressed ferroptosis by producing GSH and MUFAs [24]. Because acetylation has a major role in cellular metabolic regulation [34], the acetylation status of SLC25A22 may have important effects on mitochondrial metabolism, a topic that requires further research. In addition, we found that the inhibition of SIRT3 decreased the level of GSH following RSL3 treatment, and the forced expression of SLC25A22 led to a partial restoration of the level of GSH that resulted from SIRT3 interference of LUAD cells that were treated with RSL3. SIRT3 has many potential mitochondrial substrates. Thus, the reason for this partial response may be that SIRT3 inhibition increases the levels of ROS and Fe^2+^ in mitochondria, and LUAD cells have other mechanisms by which SIRT3 protects these cells from ferroptosis.

Despite our novel findings, there are certain limitations of this study. Firstly, although we identified K83 as the major acetylated site of SLC25A22, there are still other acetylated sites presenting in SLC25A22 since the acetylation band of SLC25A22 was not totally abolished after mutation. Because the deacetylation of SLC25A22 by SIRT3 stabilized this protein, the interplay between the acetylation and ubiquitination of SLC25A22 requires further investigation. Secondly, our results showed that the SLC25A22 and SIRT3 proteins were upregulated in LUAD cells during RSL3-induced ferroptosis; although the level of SLC25A22 was only increased at the protein level, the level of SIRT3 was increased at the mRNA and protein levels. The increased expression of SIRT3 and SLC25A22 might be part of an adaptive response used by LUAD cells to antagonize RSL3-induced ferroptosis. Our previous study demonstrated that the SIRT3 protein was upregulated during finasteride-induced ferroptosis in GBM [23]. We also found that SIRT3 functioned in the process of autophagic degradation in glioblastoma stem cells [35]. Another study showed that the Newcastle disease virus degraded SIRT3 by induction of mitophagy [36]. All of these findings indicate that the regulation of SIRT3 expression is very complex and appears to depend on the type of cell and cell status. Moreover, we found that the level of SLC25A22 was elevated during ferroptosis of LUAD cells, but Liu et al. showed that the level of SLC25A22 was decreased in four lines of PDAC cells upon RSL3 treatment [24]. The different effects of SLC25A22 during RSL3-induced ferroptosis of different tumor cells need further investigation.

## 5. Conclusions

In summary, we elucidated a novel mechanism by which SIRT3 prevented ferroptosis in LUAD cells treated with RSL3: SIRT3 deacetylates SLC25A22, and this increases its half-life. The present study is, therefore, the first to show that SLC25A22, a mitochondrial glutamate transporter, is regulated by the ubiquitin–proteasome system. In future work, we plan to study the mechanism by which SLC25A22 is degraded and elucidate its roles in regulating mitochondrial metabolism and the progression of LUAD.

## Data Availability

The raw data supporting the conclusions of this article are available on request as they are part of an ongoing study.

## Supplementary Materials

The following supporting information can be downloaded at: https://www.mdpi.com/article/10.3390/antiox14040403/s1, Figure S1. Expression of SLC25A22 in a tissue microarray (Cat No. HLugA150CS04). Figure S2. Expression of SIRT3 in a tissue microarray (Cat No. HLugA150CS04). Figure S3. RSL3 induces ferroptosis of LUAD cells. Figure S4. SLC25A22 and SIRT3 are both upregulated during erastin-induced ferroptosis of LUAD cells. Figure S5. Inhibition of SIRT3 expression or activity suppresses the expression of SLC25A22. Figure S6. Inhibition of SIRT3 activity promotes RSL3-induced ferroptosis of A549 cells in vitro. Table S1. Antibodies and Reagents. Table S2. Primers for RT-qPCR.
