# Supplementary material for: Sirtuin 3 Protects Lung Adenocarcinoma from Ferroptosis by Deacetylating and Stabilizing Mitochondrial Glutamate Transporter Solute Carrier Family 25 Member A22
Source: Antioxidants (Basel). 2025 Mar 28;14(4):403. doi: 10.3390/antiox14040403 (PMC12024224; doi:10.3390/antiox14040403)
Supplement: Supplementary file 1 [file antioxidants-14-00403-s001.zip › antioxidants-3475379-supplementary.pdf]

Supplemental materials and methods

3-TYP treatment

A549 cells were treated with DMSO or 50 µM 3-TYP for 24 h at 37 °C in an incubator with 5% CO_2. Further analyses were described in **Materials and Methods** section.

Erastin treatment

A549 and H2122 cells were treated with DMSO or erastin (20 µM and 30 µM) for 24 h at 37 °C in a 5% CO2 incubator. Further analyses were as described elsewhere in **Materials and Methods** section.

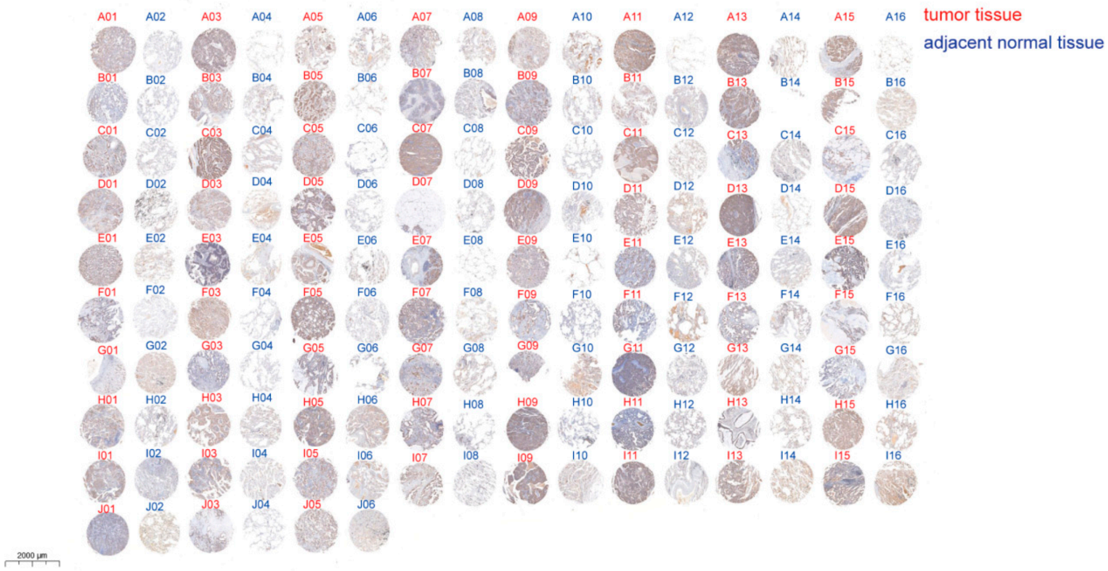

Figure S1. Expression of SLC25A22 in a tissue microarray (Cat No. HLugA150CS04).

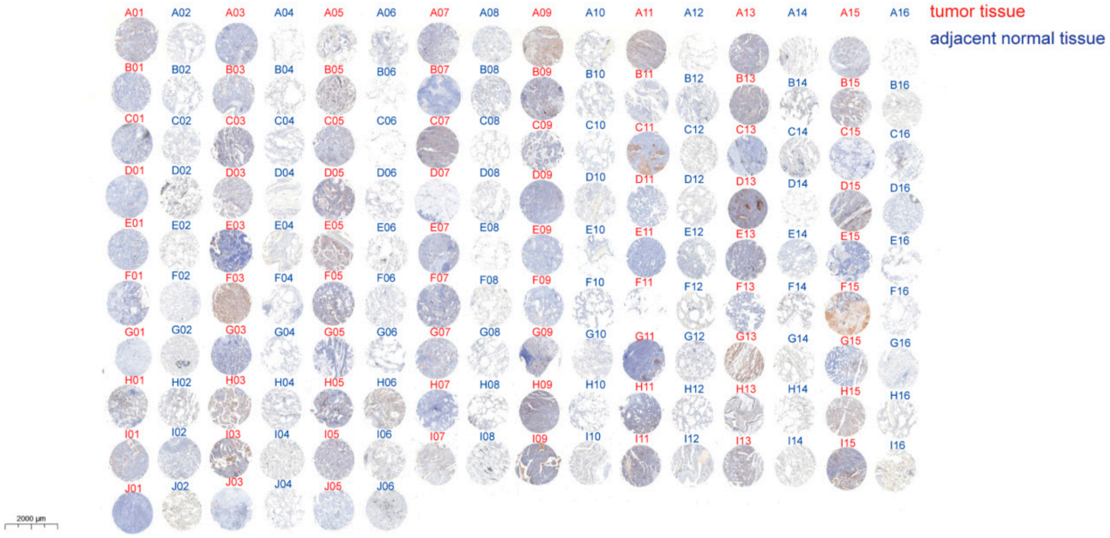

Figure S2. Expression of SIRT3 in a tissue microarray (Cat No. HLugA150CS04).

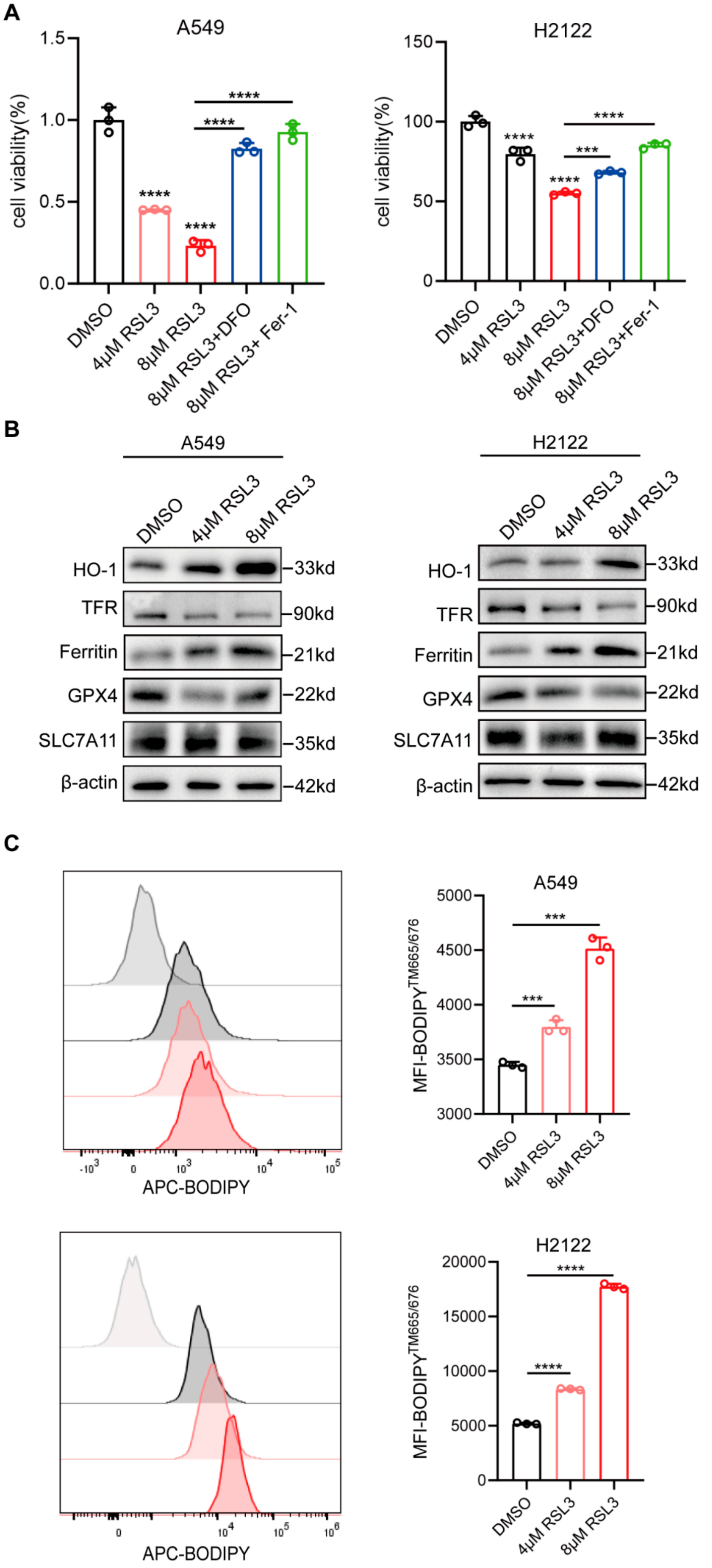
A
A549
H2122
cell viability(%)
1.5
1.0
0.5
0.0
150
100
50
0
****
***
DMSO
4μM RSL3
8μM RSL3
8μM RSL3+DFO
8μM RSL3+ Fer-1
8μM RSL3+Fer-1
B
A549
H2122
DMSO
4μM RSL3
8μM RSL3
HO-1
TFR
Ferritin
GPX4
SLC7A11
β-actin
33kd
90kd
21kd
22kd
35kd
42kd
C
APC-BODIPY
MFI-BODIPY TM665/676
5000
4500
4000
3500
3000
20000
15000
10000
5000
0

Figure S3. RSL3 induces ferroptosis of LUAD cells. **A-C** A549 or H2122 cells were treated with DMSO (control) or RSL3 (4 μM and 8 μM) for 12 or 24 hours. **A** Cell viability was assessed using the CCK-8 assay. The concentration of deferoxamine (DFO) used was 100 μM for A549 cells and 5 μM for H2122 cells; The concentration of Ferrostatin-1 (Fer-1) used was 1 μM for A549 cells and 0.2 μM for H2122 cells. **B** Protein expression levels of HO-1, TFR1, Ferritin, GPX4, and SLC7A11 were detected by Western blot. **C** Intracellular lipid peroxidation levels were measured by flow cytometry using the BODIPY™ 665/676 probe. Data are presented as mean ± SD. **$p<0.01$, ***$p<0.001$, ****$p<0.0001$. DFO, deferoxamine; LPO, lipid peroxidation; HO-1, heme oxygenase 1; TFR1, transferrin 1; GPX4, Glutathione Peroxidase 4; SLC7A11, solute family 7 member 11.

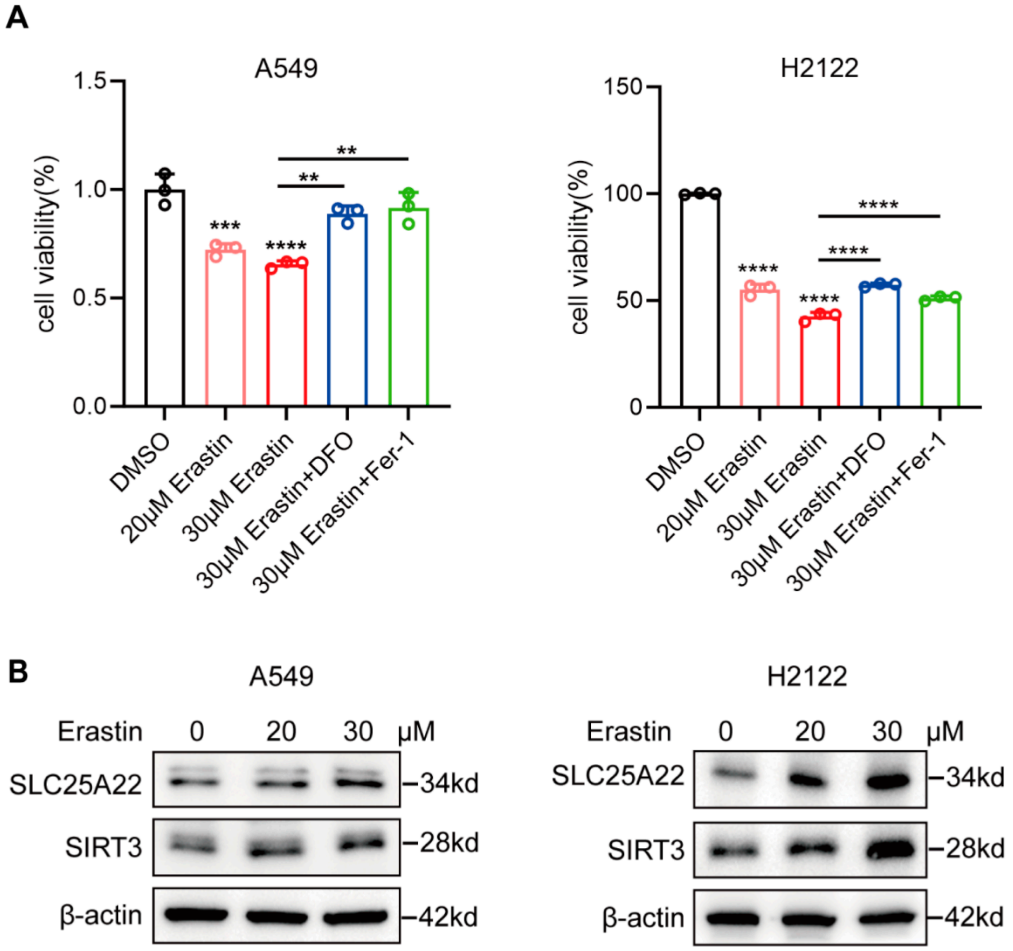

Figure S4 SLC25A22 and SIRT3 are both upregulated during erastin-induced ferroptosis of LUAD cells. A549 and H2122 cells were treated with DMSO or erastin (20 μM or 30 μM) for 24 hours. A Cell viability was assessed using the CCK-8 assay. The concentration of deferoxamine (DFO) used was 100 μM for A549 cells and 5 μM for H2122 cells. The concentration of Ferrostatin-1 (Fer-1) used was 1 μM for A549 cells and 0.2 μM for H2122 cells. B The protein expression levels of SIRT3 and SLC25A22 were detected by Western blot. Data are presented as mean ± SD. **$p < 0.01$, ***$p < 0.001$, ****$p < 0.0001$.

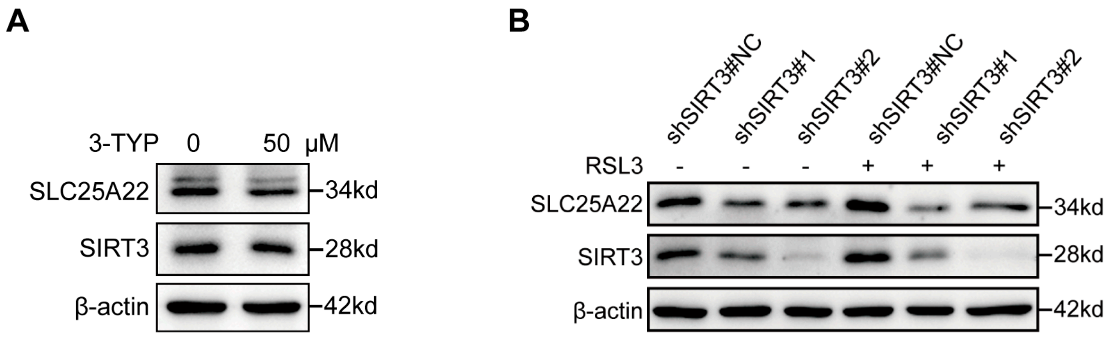

Figure S5 Inhibition of SIRT3 expression or activity suppresses the expression of SLC25A22. A Total proteins were extracted from A549 cells treated with DMSO or 3-TYP (50 μM) for 24 hours. The expression levels of SLC25A22 were detected by Western blot. **B** Total proteins were extracted from A549 cells transfected with shNC, shSIRT3#1, or shSIRT3#2 and treated with DMSO or RSL3 (8 μM) for 12 hours. The expression levels of SLC25A22 were detected by Western blot.

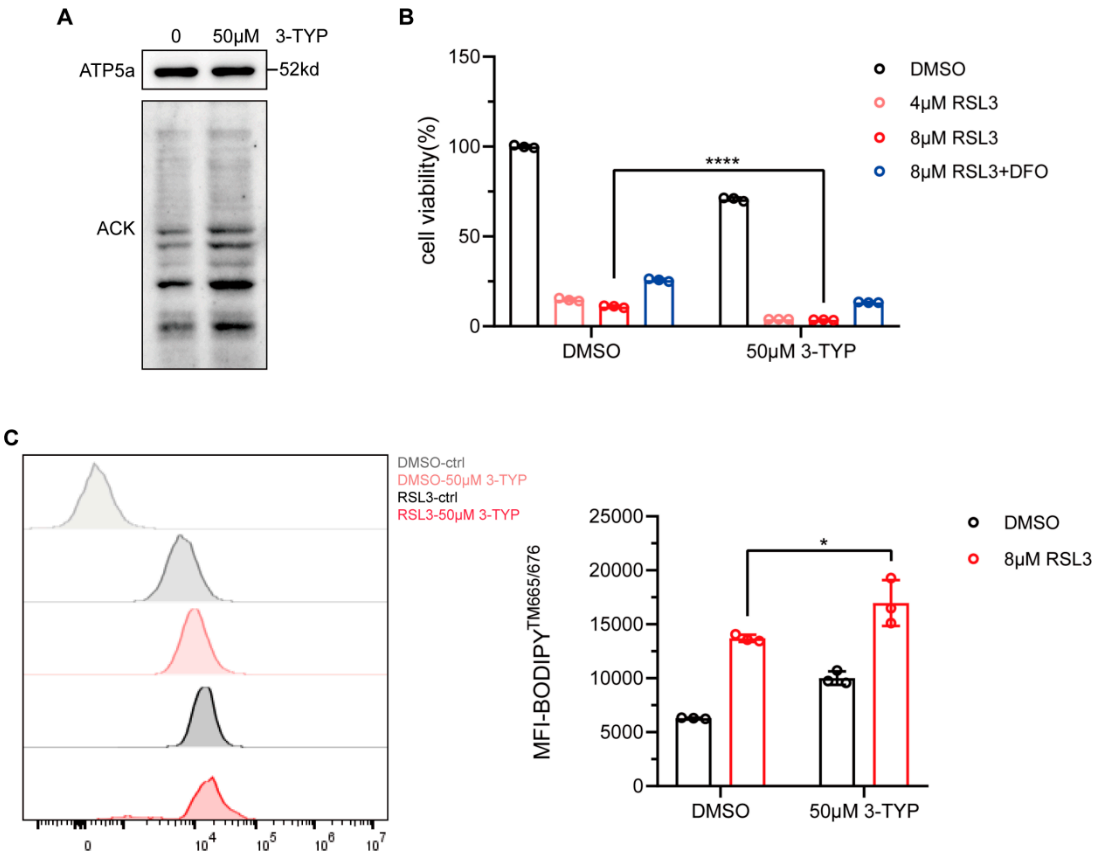

Figure S6. Inhibition of SIRT3 activity promotes RSL3-induced ferroptosis of A549 cells *in vitro*. **A** 3-TYP inhibited SIRT3 deacetylation activity in A549 cells. **B** 3-TYP promoted RSL3-induced ferroptosis of A549 cells, and this was partially rescued by DFO. **C** 3-TYP promoted the accumulation of LPO in A549 cells. *p<0.05, ****p<0.0001.

Table S1 Antibodies and Reagents

Reagents and Antibodies	Identifier	Source
Anti-GC-1 Antibody	ab137614	Abcam
Anti-Ferritin Antibody	ab75973	Abcam
Anti-Glutathione Peroxidase 4 antibody	ab125066	Abcam
Anti-ATP5A antibody	ab110273	Abcam
Heme Oxygenase 1 (HO-1) Rabbit Monoclonal Antibody	AF1333	Beyotime
Anti-SIRT3 Antibody	5490S	Cell Signaling Technology
xCT/SLC7A11 (D2M7A) Rabbit mAb	12691	Cell Signaling Technology
Acetylated-Lysine Antibody	9441S	Cell Signaling Technology
Ubiquitin (P4D1) Mouse mAb	3936S	Cell Signaling Technology
Transferrin Receptor Antibody	13-6890	Invitrogen
Beta Actin Mouse Monoclonal Antibody	60008-1-Ig	Proteintech
HA-Tag Antibody	sc-7392	Santa Cruz Biotechnology
Monoclonal ANTI-FLAG® M2	F1804	Sigma-Aldrich
Hieff Mut™ Site-Directed Mutagenesis Kit	11003ES10	YEASEN
Phanta Max Super-Fidelity DNA Polymerase	P505	vazyme
Thermo Scientific™ FastDigest DpnI	FD1704	ThermoFisher
DNA Ligation Kit Ver.2.1	6022	Takara
RSL3	S8155	Selleck
DFO	S6849	Selleck
3-TYP	S8628	Selleck
Cycloheximide	S7418	Selleck
MG132	S2619	Selleck
Chloroquine	S6999	Selleck
Protease Inhibitor Cocktail	B14001	Selleck
TRNzol Universal Reagent	DP424	TIANGEN
Cell Counting Kit-8	CK04	DOJINDO
Mito-FerroGreen	M489	DOJINDO
BODIPY 665/676 (Lipid Peroxidation Sensor)	B3932	Invitrogen
MitoSOX Red	M36008	Invitrogen
TRNzol Universal Reagent	DP424	TIANGEN
PrimeScript™ RT Master Mix	RR036B	Takara
ChamQ Universal SYBR qPCR Master Mix	Q711-03	vazyme
Hieff Trans® Liposomal Transfection Reagent	40802ES03	YEASEN
Hieff Mut^{TM} Site-Directed Mutagenesis Kit	11003ES10	YEASEN
GSH and GSSG Assay Kit	S0053	Beyotime
Anti-HA Magnetic Beads	HY-K0201	medchemexpress
M2-Flag affinity gel	A2220	Sigma-Aldrich
rProtein A Magarose Bead	SM003005	smart-lifesciences

Table S2 Primers for RT-qPCR

Gene	Primer Sequence	
18s	Forward Sequence	GAAACGGCTACCACATCC
	Reverse Sequence	CACCAGACTTGCCCTCCA
β-actin	Forward Sequence	CCTGGCACCCAGCACAAT
	Reverse Sequence	GGGCCGGACTCGTCATAC
SIRT3	Forward Sequence	CCCCAAGCCCTTTTTCACTTT
	Reverse Sequence	CGACACTCTCTCAAGCCCA
SLC25A22	Forward Sequence	GTCAACGAGGACACCTACTCTG
	Reverse Sequence	GGAAGTAGACCACCTGTGCGAT
SLC25A22^{K83R}	Forward Sequence	AAGGCCATCAGGCTGGCAGCCAACGACTTCTTC
	Reverse Sequence	TGCCAGCCTGATGGCCTTCTCGGGGGTGACGAG
